# Effect of Glutamic Acid and 6-benzylaminopurine on Flower Bud Biostimulation, Fruit Quality and Antioxidant Activity in Blueberry

**DOI:** 10.3390/plants12122363

**Published:** 2023-06-18

**Authors:** María Itzel Pérez-León, José Antonio González-Fuentes, Luis Alonso Valdez-Aguilar, Adalberto Benavides-Mendoza, Daniela Alvarado-Camarillo, Carlos Estuardo Castillo-Chacón

**Affiliations:** 1 Departamento de Horticultura, Universidad Autónoma Agraria Antonio Narro, Saltillo 25315, Coahuila, Mexico; maria.perez@colpos.mx (M.I.P.-L.); abenmen@gmail.com (A.B.-M.); 2 Departamento de Ciencias del Suelo, Universidad Autónoma Agraria Antonio Narro, Saltillo 25315, Coahuila, Mexico; daniela.alvaradoc@uaaan.edu.mx; 3 Consultores Técnicos en Producción Agrícola, Guadalajara 45645, Jalisco, Mexico; carlosecastillo65@gmail.com

**Keywords:** antioxidants, biostimulant, flower buds, nutraceutical quality

## Abstract

Blueberry is a highly demanded and consumed fruit due to its beneficial effects on human health, because of its bioactive compounds with a high antioxidant capacity. The interest in increasing the yield and quality of blueberries has led to the application of some innovative techniques such as biostimulation. The objective of this research was to assess the effect of the exogenous application of glutamic acid (GLU) and 6-benzylaminopurine (6-BAP) as biostimulants on flower bud sprouting, fruit quality, and antioxidant compounds in blueberry cv. Biloxi. The application of GLU and 6-BAP positively affected bud sprouting, fruit quality, and antioxidant content. The application of 500 and 10 mg L^−1^ GLU and 6-BAP, respectively, increased the number of flower buds, while 500 and 20 mg L^−1^ generated fruits with higher content of flavonoids, vitamin C, and anthocyanins and higher enzymatic activity of catalase and ascorbate peroxidase enzymes. Hence, the application of these biostimulants is an effective way to enhance the yield and fruit quality of blueberries.

## 1. Introduction

Blueberry (*Vaccinium corymbosum* L.) is characterized by low flower bud production and has long periods of production and staggered ripening of the fruit within the plant, implying that several harvests are carried out, and, consequently, production costs rise [1]. Currently Mexico is seeking to become one of the main blueberry producers; however, to achieve this goal, it is necessary to implement sustainable and environmentally friendly production techniques [2]. These techniques should allow timely and uniform flowering to concentrate fruit production early in the season to take advantage of the high prices in the market. On the other hand, an alternative technique that has worked out in other fruit trees to manipulate fruit production is the use of biostimulants [3], which can stimulate plant growth and development and improve nutrition, quality, and resistance to different types of stress when exogenously applied at low concentrations [4,5,6]. Amino acids such as glutamate (GLU) and phytohormones such as 6-benzylaminopurine (6-BAP) are considered biostimulants according to the classifications proposed by du Jardin [5], the European Union [7] and the Mexican standard NOM-182-SSA1-2010 [8].

Glutamate is one of the most abundant amino acids, and it can exist as free GLU or as GLU bound with other amino acids to form peptides [9]. It plays an important role in plant germination, growth, and development [10,11,12]. The application of GLU is reported to induce the sprouting of vegetative and reproductive buds, increase chlorophyll concentration, and improve the quality of fruits, including weight, size, firmness, and the concentration of citric acid [13,14,15]. It affects pollination and fruit set and induces the production of secondary metabolites [16,17,18,19] and the expression of genes related to defense and stress responses [20,21,22,23].

Cytokinins such as 6-BAP are plant hormones involved in growth and development, the regulation of cell division processes, the delay in senescence, and the regulation of apical dormancy [24,25]. It was reported that the application of 6-BAP in selected crops favors the production of buds [26,27] and the generation of roots and flowers [28,29], in addition to the removal of reactive oxygen species [30,31,32,33].

As worldwide public health awareness and the demand for functional foods with multitudinous health benefits have increased [34], blueberries have gained popularity in recent years due to their high content of bioactive compounds with high antioxidant capacity. They have a wide range of pharmacological effects, including anticancer [35], antioxidant [36], anti-inflammatory [37], and anti-obesity [38] effects and the prevention and treatment of degenerative and cardiovascular diseases [39].

In this context, the main objective of this study was to assess and evaluate the effects of the exogenous application of GLU and 6-BAP as biostimulants on flower bud sprouting, fruit quality, and antioxidant compounds in blueberry cv. Biloxi.

## 2. Results

### 2.1. Number of Buds and Fruit Quality

**Figure 1:**
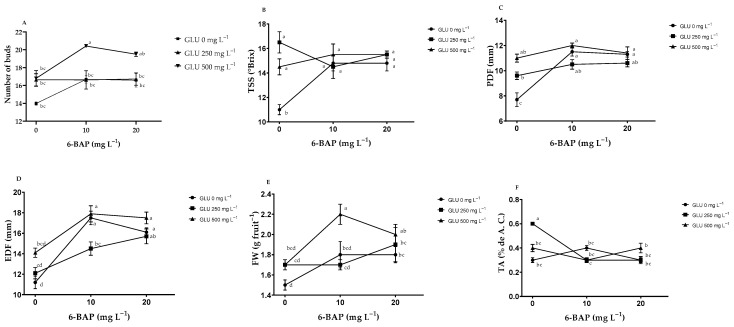
Effect of the interaction of the biostimulants glutamate (GLU) and 6−benzylaminopurine (6−BAP) in blueberry (*Vaccinium corymbosum* L.) Biloxi: (**A**) number of buds, (**B**) Total soluble solids (TSS), (**C**) polar diameter of fruit (PDF), (**D**) equatorial diameter of fruit (EDF), (**E**) fruit weight (FW), (**F**) titratable acidity (TA). Bars represent the standard error of the mean. Different letters indicate significant difference (Tukey’s, *p* ≤ 0.05).

**Interaction GLU and 6**-**BAP:** Plants that received an application of GLU*6-BAP at 500–10 mg L^−1^ and 500–20 mg L^−1^ showed a greater number of buds per stem, surpassing 46% and 40%, respectively, than that of the control plants (Figure 1A, Table 1). The lowest production of TSS occurred in those plants with no applied biostimulants; however, TSS increased up to 38% when GLU and 6-BAP were applied (Figure 1B). The polar and equatorial diameters of the fruits in plants treated with GLU at 500 mg L^−1^ increased with the addition of 6-BAP at 10 mg L^−1^; a similar effect was observed in plants when GLU was not applied (Figure 1C, D). Plants treated with GLU at 500 mg L^−1^ showed increased fruit weight when 6-BAP at 10 mg L^−1^ was added; however, when 6-BAP was increased to 20 mg L^−1^, fruit weight tended to decrease (Figure 1E). The application of GLU 250 mgL^−1^ caused a significant increase of 80% in TA when 6-BAP was not added; however, TA decreased when 6-BAP was at 10 mg L^−1^ (Figure 1F).

**Effects of GLU and 6-BAP:** The application of GLU increased bud sprouting and fruit quality (Table 1). Plants treated with GLU 500 mg L^−1^ showed a 23% increase in the number of buds per stem, while for the total soluble solids (TSS), polar diameter, equatorial diameter, and fruit weight, they exhibited an increase of 15%, 12%, 16%, and 15%, respectively, compared to the control plants (Table 1). The application of 6-BAP also increased the number of buds, polar diameter, equatorial diameter, and fruit weight, generating significant increases of 15%, 19%, 32%, and 14%, respectively, when compared to the control plants (Table 1). The TSS in fruits from plants treated with 6-BAP was not significantly different compared to the control (Table 1).

### 2.2. Nonenzymatic Antioxidants in Fruits

**Figure 2:**
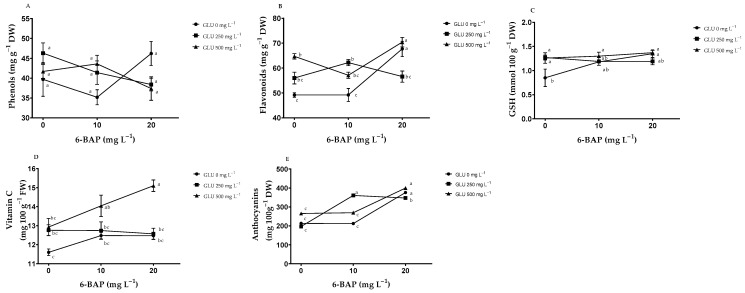
Effect of the interaction of the biostimulants glutamate (GLU) and 6-benzylaminopurine (6-BAP) on the content of nonenzymatic antioxidants in blueberry (*Vaccinium corymbosum* L.) fruits: (**A**) phenols, (**B**) flavonoids, (**C**) reduced glutathione (GSH), (**D**) vitamin C, (**E**)anthocyanin. Dry weight (DW), fresh weight (FW). Bars represent the standard error of the mean. Different letters indicate significant difference (Tukey’s, *p* ≤ 0.05).

**Interaction between GLU and 6**–**BAP:** The interaction did not present a significant effect on the content of phenols in the fruit (Figure 2A); however, there was a significant increase in the content of flavonoids in fruit in plants when GLU at 500 mgL^−1^ was applied in synergy with 6-BAP 20 mgL^−1^ (Figure 2B). The concentration of reduced glutathione (GSH) in fruit increased as the concentration of GLU and 6-BAP increased (Figure 2C). The application of GLU 500 mg L^−1^ in synergy with 6-BAP 20 mg L^−1^ presented a higher vitamin C concentration, exceeding by 30% that obtained by the fruit from control plants (Figure 2D). In plants when GLU 500 mg L^−1^ was applied, the anthocyanin content increased as the dose of 6-BAP was increased, and a similar trend was observed in plants when GLU was not applied (Figure 2E).

**Effects of GLU and 6-BAP:** The phenols in fruit were not influenced by the application of the treatments (Table 2). In contrast, the content of flavonoids, GSH, and vitamin C increased by 16%, 14% and 17%, respectively, with the application of GLU at 500 mg L^−1^. The anthocyanin content did not show differences between plants treated with GLU at 250 and 500 mg L^−1^; however, when compared to control plants, there was an increase of 15%.

The application of 6-BAP at 20 mg L^−1^ increased the content of flavonoids, GSH, vitamin C, and anthocyanins by 15%, 15%, 9%, and 66%, respectively (Table 2).

### 2.3. Nonenzymatic Antioxidants in Leaves

**Figure 3:**
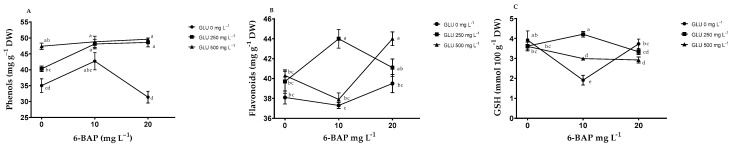
Effect of the interaction of the biostimulants glutamate (GLU) and 6-benzylaminopurine (6-BAP) on nonenzymatic antioxidant content in blueberry *(Vaccinium corymbosum* L.) leaves: (**A**) phenols, (**B**) flavonoids, (**C**) reduced glutathione (GSH). Dry weight (DW). Bars represent the standard error of the mean. Different letters indicate significant difference (Tukey’s, *p* ≤ 0.05).

**Interaction GLU and 6-BAP:** The interaction of GLU and 6-BAP generated modifications in the content of the nonenzymatic antioxidants in leaves (Figure 3). The concentration of the phenols in the leaves increased in plants when GLU 250 and 500 mg L^−1^ were applied in synergy with 6-BAP 10 and 20 mg L^−1^ (Figure 3A), while the flavonoids increased by 16% with the application of 500–20 mg L^−1^ (Figure 3B). The 250–10 mg L^−1^ treatment caused a 7% increase in GSH compared to the control (Figure 3C).

**Effects of GLU and 6-BAP:** The application of GLU and 6-BAP generated modifications in the content of the nonenzymatic antioxidants in leaves (Table 3). Both concentrations of GLU increased the content of the phenols in the leaves, exceeding that of the control by up to 34%, while the content of the flavonoids presented an average increase of 7%. Both concentrations of 6-BAP induced a decrease in GSH of up to 18% in reference to the control plants.

### 2.4. Photosynthetic Pigments

**Figure 4:**
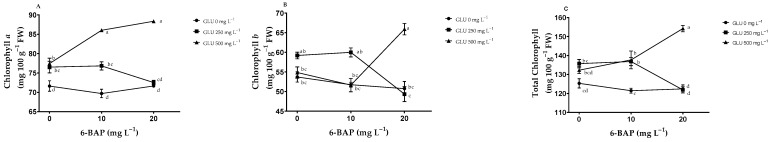
Effect of the interaction of the biostimulants glutamate (GLU) and 6-benzylaminopurine (6-BAP) on photosynthetic pigment content: (**A**) chlorophyll *a*, (**B**) chlorophyll *b*, (**C**) total chlorophyll, fresh weight (FW). Bars represent the standard error of the mean. Different letters indicate significant difference (Tukey’s, *p* ≤ 0.05).

**Interaction between GLU and 6-BAP:** Significant interactions in photosynthesis pigments were obtained owing to the application of different levels of glutamic acid and 6-benzylaminopurine. The interaction of GLU*6-BAP at concentrations of 500 and 20 mg L^−1^ showed increases of 23%, 22%, and 23% in chlorophyll *a* and *b* and total chlorophyll, respectively (Figure 4).

**Effects of GLU and 6-BAP:** Chlorophyll (*a, b*, and total) showed significant effects due to the assessed biostimulants (Table 4). Chlorophyll *a* and *b* and total chlorophyll increased by 18%, 10%, and 15%, respectively, due to GLU applications, when compared to control plants. Regarding the application of 6-BAP, the concentration of chlorophyll *a* increased by 3%, while chlorophyll *b* and total chlorophyll did not show any significant effect compared to control plants.

### 2.5. Enzymatic Antioxidants in Fruits

**Figure 5:**
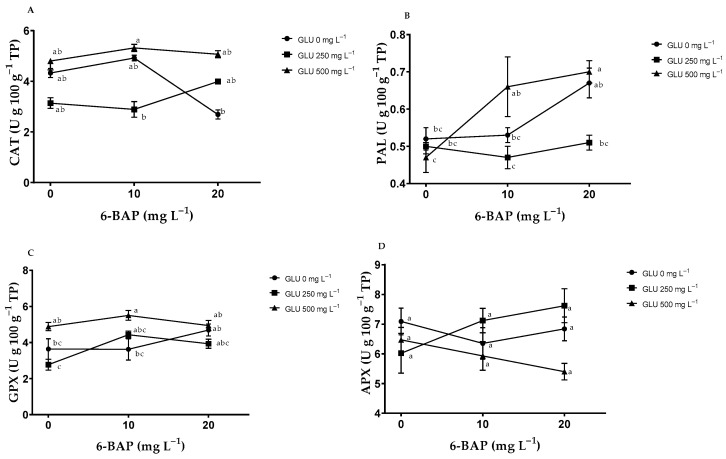
Effect of the interaction of biostimulants glutamate (GLU) and 6-benzylaminopurine (6-BAP) on enzymatic antioxidant activity in blueberry (*Vaccinium corymbosum* L.) fruits: (**A**) Catalase (CAT), (**B**) phenylalanine ammonia lyase (PAL), (**C**) glutathione peroxidase (GPX), (**D**) ascorbate peroxidase (APX). Bars represent the standard error of the mean. Different letters indicate significant difference (Tukey’s, *p* ≤ 0.05).

**Interaction between GLU and 6-BAP:** The interaction of GLU*6-BAP at concentrations of 500 and 10 mg L^−1^ induced higher CAT activity; however, it was not significantly different from that of the control (Figure 5A). GPX activity was higher in the 500–10 mg L^−1^ treatment (Figure 5C). The application of the treatments did not influence the enzymatic activity of APX (Figure 5D).

**Effects of GLU and 6-BAP:** GLU modified the activity of CAT and GPX in the fruit (Table 5). The concentration of GLU 500 mg L^−1^ increased the activity of CAT and GPX by 27% and 28%, respectively, in relation to the control. The application of GLU 250 mg L^−1^ caused a 20% decrease in PAL compared to that of the control, and there was also a 14% decrease in APX enzymatic activity when GLU 500 mg L^−1^ was applied compared to GLU 250 mg L^−1^, which caused higher activity. The application of 6-BAP did not modify the enzymatic activity of CAT and APX; however, at 20 mg L^−1^, it increased the activity of PAL and GPX by 26% and 20%, respectively.

### 2.6. Enzymatic Antioxidants in Leaves

**Figure 6:**
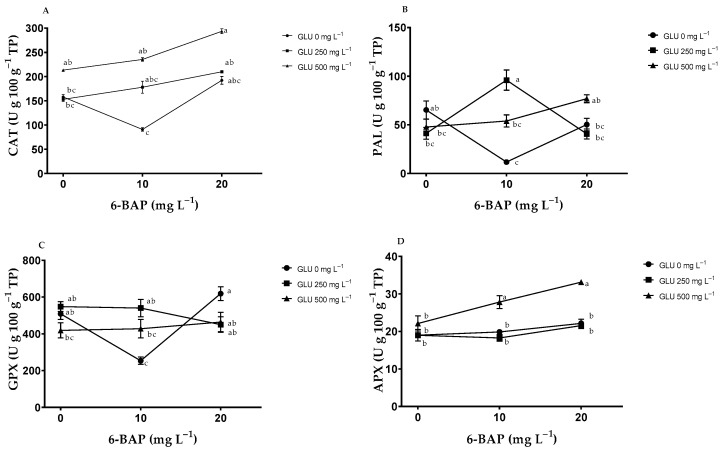
Effect of the interaction of biostimulants glutamate (GLU) and 6-benzylaminopurine (6-BAP) on the enzymatic activity in blueberry (*Vaccinium corymbosum* L.) leaves: (**A**) Catalase (CAT), (**B**) phenylalanine ammonia lyase (PAL), (**C**) glutathione peroxidase (GPX), (**D**) ascorbate peroxidase (APX). Bars represent the standard error of the mean. Different letters indicate significant difference (Tukey’s, *p* ≤ 0.05).

**Interaction GLU and 6-BAP:** The CAT and APX activities showed positive effects with the interaction of GLU and 6-BAP at 500 and 20 mg L^−1^, respectively, increasing by 86% and 74%, respectively, compared to control plants (Figure 6A,D). The highest PAL activity occurred in plants treated with GLU at 250 mg L^−1^ with added 6-BAP 10 mg L^−1^ (Figure 6B). The highest GPX activity occurred in plants when 6-BAP was applied with no GLU (Figure 6C).

**Effects of GLU and 6-BAP:** The enzymatic activity in blueberry leaves was affected by the applied treatments (Table 6). GLU had a positive effect on CAT and APX, generating increases of 68% and 36%, respectively, when applied at 500 mg L^−1^, while GLU at 250 mg L^−1^ did not cause any significant effect compared to control plants. Regarding PAL and GPX, there was no effect caused by either concentration of GLU. With respect to the application of 6-BAP at 20 mg L^−1^, increases of 33% and 28% of CAT and APX, respectively, were observed, while, at this concentration, PAL and GPX were not different from control plants.

## 3. Discussion

### 3.1. Number of Buds and Fruit Quality

Flowering is one of the most crucial stages in the plant life cycle, since it represents the transformation from the vegetative phase to the reproductive phase [40]. This stage commences with the induction of floral buds, followed by the differentiation of primordia and finally the maturation of the floral organs [41,42]. An increase in the number of flower buds and quantity of flowers induces greater fruit formation, which could be associated with a higher fruit yield [43,44]. As expected, GLU and 6-BAP (Table 1) increased bud sprouting in blueberry, which concurs with reports by El-Metwally et al. [45] showing that 20 mg L^−1^ GLU increased the number of branches and fruits per plant in peanut, whereas the application of 5 mM (735 mg L^−1^) GLU in sunflower improved the morphological characteristics, root length, plant height, and number of flowers [46]. Regarding the beneficial effects of 6-BAP, Li et al. [27] and Zhang et al. [47] reported that the application of 300 and 30 mg L^−1^ on apple and mulberry, respectively, increased the growth and the number of shoots and buds.

Fruit quality parameters such as fruit weight, size, TSS, and acidity content [48] were improved by the biostimulant application; similar results were reported by Ariza Flores et al. [49], indicating an increase in citric acid in lime fruits with the application of GLU at 0.45 kg ha^−1^. The total soluble solids observed in the present study ranged between 11 and 16.5° Brix, with acidity lower than 0.7%; these parameters coincide with the quality standards reported by Madrid and Beaudry [50], stating that the acidity of blueberry fruits should not exceed 0.7% and that °Brix must be higher than 10%. In addition, the size of the fruits harvested, except for those of the control, were rated as large, according to the quality protocol for fresh blueberries published by FAO [51], which classifies the size of the fruit according to the equatorial diameter as small (6–8 mm), medium (9–11 mm), and large (≥12 mm), with the exception of the control. Similar findings were reported with BAP applications that increased the quality and size of the fruit [52]; additionally, the application of 100 mg L^−1^ BAP increased the fruit size and yield in Duke and Bluecrop blueberries [53]. Furthermore, Abdelgadir et al. [54] reported an increased number of flowers per plant, number of fruits per cluster, and weight and size of *Jatropha curcas* fruits with the application of 6-BAP at 3 mM (676 mg L^−1^).

### 3.2. Nonenzymatic Antioxidants

The interest in producing and marketing blueberries is related to their high content of bioactive compounds such as phenols, flavonoids, and anthocyanins, among others, which are beneficial to human health [55]. The beneficial effects of these compounds are mainly due to their antioxidant properties and free radical scavenging capacity in the human body [56]. However, our results showed that applying GLU and 6-BAP caused further increases in the activity of nonenzymatic antioxidants such as flavonoids, GSH, vitamin C, and anthocyanins, thus improving the nutraceutical quality of blueberry fruits. The findings reported here (Table 2 and Table 3) agree with those of El-Metwally et al. [45], who reported that GLU increased the content of flavonoids and phenols in peanut seeds and leaves. The exogenous application of GLU at different concentrations promoted the accumulation of anthocyanins in litchi fruits and in the leaves of apple, pear, and peach [57,58,59,60]. An increase in the content of the total phenols in onion bulbs and an increase in the content of the flavonoids in the leaves and roots of *Crataegus pinnatifida* were reported when applying GLU [61,62]. In mulberry leaves and cucumber fruits, increases in the flavonoids content and total phenols, respectively, were reported when applying 6-BAP [47,63].

### 3.3. Photosynthetic Pigments

Several authors noted the positive effect of GLU on photosynthetic efficiency and chlorophyll concentration. Our findings indicate that in blueberry there is an increase in chlorophyll *a* by the application of 6-BAP and a significant increase in chlorophyll *a* and *b* and total chlorophyll by the interaction of GLU*6-BAP at higher concentrations (Table 4); these results agree with those reported by El-Metwally et al. [45], as the application of 20 mg L^−1^ GLU increased the content of chlorophyll *a* and *b* and total chlorophyll in peanuts. In contrast, Franzoni et al. [64] and Wang et al. [33] reported that applying GLU and 6-BAP had no positive effect on the chlorophyll content in and yield of lettuce and maize. 

### 3.4. Enzymatic Antioxidants

During the process of establishment, development, and growth, plants face severe conditions causing stress and increased production of reactive oxygen species (ROS) [47]. ROS are present even when plants grow under optimal conditions [65]. ROS, including hydrogen peroxide (H_2_O_2_), hydroxyl radical (OH^−^), superoxide anion (O_2_^−^), and singlet oxygen (O_2_), are byproducts of metabolic processes [66]. Excessive ROS production leads to lipid peroxidation, membrane injury, enzyme inactivation, inhibition of photosynthesis, respiration, plant growth, and secondary metabolite production [67]. Plants have developed defense mechanisms capable of eliminating ROS and preventing oxidative damage, which include antioxidant enzymes such as superoxide dismutase (SOD), peroxidase (POD), CAT, APX, and glutathione reductase (GR) and nonenzymatic antioxidants such as ascorbate (AsA) and GSH [68,69]. According to these arguments, the increased enzymatic antioxidant concentrations in treated plants observed in the present study (Table 5 and Table 6) suggest the possibility of inducing blueberry plants to produce antioxidants in larger quantities to protect themselves against increasingly adverse environmental conditions.

Various authors reported a decrease in reactive oxygen species and lipid peroxidation through applications of GLU and 6-BAP that resulted in increased enzymatic activity [70]. The results reported by Chen et al. [67] and Yang et al. [31] showed that 6-BAP increased the enzymatic activity of CAT and APX. Other studies reported that GLU favored higher APX and CAT activity in the leaves and roots of sunflower plants, while Farid et al. [46] reported higher CAT activity in soybean [71].

Although PAL is not an antioxidant, it is a key enzyme in the phenylpropanoid pathway, and it catalyzes the conversion of L-phenylalanine into trans-cinnamic acid, which is the precursor of a variety of phenolic compounds with structural and defense functions, such as lignin, flavonoids, and coumarins [72]. The results observed in the present study partially agree with those of Cui et al. [62], QiaoZhen et al. [73], Teixeira et al. [71], and Zhang et al. [47], who reported increases in PAL caused by the application of GLU and 6-BAP. Increases in PAL activity can be induced by applying exogenous agents, including some hormones [74].

The effectiveness of GLU and 6-BAP treatments largely depends on the species, concentration, timing, and method of application; the doses reported by various researchers presented null or toxic effects when applied to other species [64,65].

## 4. Materials and Methods

### 4.1. Study Area

The study was carried out in a tunnel-type greenhouse in the Department of Horticulture at the Antonio Narro Autonomous Agrarian University in Saltillo, Coahuila, Mexico, which is located between the geographic coordinates of 25°22′ north latitude and 101°02′ west longitude and at an altitude of 1742 m above sea level.

### 4.2. Vegetal Material

Two-year-old Biloxi blueberry plants were grown in 30 L containers with coconut fiber as growing medium. Mineral nutrition was modified according to the phenological stage of the plants (Table 7), and it was applied through a drip irrigation system.

### 4.3. Experimental Design and Treatments

The experiment was established as a completely randomized factorial design with nine treatments (Table 8) and six replicates each; the treatments consisted of three different concentrations of GLU and three of 6-BAP plus the interaction of both factors. GLU (99%, Sigma Aldrich, St. Louis, MO, USA) was dissolved in distilled water, while 6-BAP (99%, Sigma Aldrich, St. Louis, MO, USA) was dissolved in 1 mL of ethanol and subsequently diluted with distilled water to obtain the desired concentrations. The treatments were applied weekly (for eight weeks) by drenching after pruning.

### 4.4. Fruit Quality

Samples of 50 ripe fruits from each treatment and replication were taken and evaluated. Total soluble solids (°Brix) were evaluated by placing a drop of fruit juice on the lens of an analog refractometer (ATAGO, MASTER-alfa, USA). The polar and equatorial diameters of the fruit (mm) were measured with a digital caliper (STEREN model HER-411, MX). Fruit weight (g) was determined with a balance (TJ model MH-500, MX).

#### Titrimetric Methods

Titratable acidity (% citric acid) was determined by titrimetry, according to Capocasa et al. [75]. Then, 20 g fresh fruit were weighed and homogeneously macerated, the mixture was filtered with a sterile gauze, 10 mL of the macerate were taken, and five drops of phenolphthalein were added and titrated with sodium hydroxide (NaOH, 0.1 N) until a pinkish coloration was obtained. The quantification of titratable acidity was determined using Equation S1. 

Vitamin C (mg 100 g^−1^ fresh weight) was determined by the titration method with 2,6 dichlorophenolindophenol [76]. Then, 20 g of fresh fruit were weighed and macerated in a mortar with 10 mL of hydrochloric acid (HCl) 2%; 100 mL of distilled water were added and filtered through sterile gauze; and then a 10 mL aliquot was taken and titrated with 2–6 dichlorophenolindophenol until a pinkish color was obtained. The quantification of vitamin C was determined using Equation S2. 

### 4.5. Sample Preparation for Biochemical Analysis.

Ripe fruits and leaves were collected from each treatment, which were freeze-dried (FreeZone2.5-L Benchtop Free Dry System, LABCON, Kansas, MO, USA) and ground with a mortar to later carry out the subsequent analyses. Fruits were sampled when they had completely developed a blue color and were free of damage and lesions.

#### 4.5.1. Nonenzymatic Antioxidants

The content of total phenols was determined according to Yu and Dahlgre [77], and the calibration curve was performed using gallic acid (Figure S1). 

The flavonoids content was determined according to Arvouet-Grand et al. [78], and the calibration curve was performed using catechin as a standard (Figure S2). 

Reduced glutathione (GSH) was determined by reaction with 5,5 dithio-bis-2 nitro benzoic acid (DTNB), according to the technique reported by Xue et al. [79]. Then, 0.480 µL of enzyme extract, 2.2 mL of dibasic sodium phosphate (Na_2_HPO_4_ at 0.32 M), and 0.32 mL of DTNB dye (1 mM) were placed in a test tube. Subsequently, the mixture was vortexed and read in a UV–Vis spectrophotometer at 412 nm. The calibration curve was performed using reduced glutathione as a standard (Figure S3).

Anthocyanins were quantified by differential pH, according to the technique described by Giusti and Wrolstad [80]. Then, 50 mg of lyophilized tissue were weighed, and 5 mL of ethanol acidified with 1% hydrochloric acid (HCl) were added. The mixture was centrifuged at 4000 rpm for 15 min at 0°. The reaction mixture consisted of 2 phases: in phase 1, 400 µL of extract was mixed with 1600 µL of 0.025 M potassium chloride KCl (pH 1.0); in phase 2, 400 µL of extract was mixed with 1600 µL of 0.4 M sodium acetate chloride (pH 4.5). The absorbance of both samples was read at 520 and 700 nm. The quantification of anthocyanins was determined using Equation S3. 

#### 4.5.2. Enzymatic Antioxidants

Catalase (CAT, EC 1.11.1.6) was determined according to Dhindsa et al. [81], and the calibration curve was performed using hydrogen peroxide (Figure S4). Glutathione peroxidase (GPX, EC 1.11.1.9) was determined by the methodology of Flohé et al. [82], and the calibration curve was performed using reduced glutathione (Figure S5). Phenylalanine ammonium lyase (PAL, EC 4.3.1.5) was determined, according to the methodology of Sykłowska-Baranek et al. [83], and the calibration curve was performed using transynamic acid (Figure S6). Ascorbate peroxidase (APX, EC 1.11.1.11) was determined, according to what was established by Elavarthi and Martin [84], and the calibration curve was performed using ascorbic acid (Figure S7). 

### 4.6. Photosynthetic Pigments

The content of chlorophyll *a*, chlorophyll *b*, and total chlorophyll were determined in leaves, according to the methodology reported by Arnon [85] and Munira et al. [86]. Then, 50 mg of lyophilized tissue were weighed, 10 mg of magnesium carbonate and 2 mL of 90% acetone were added, and then it was centrifuged for 5 min at 10,000 rpm at 4 °C; the supernatant was taken and read in a spectrophotometer at 645 and 663 nm. The results were expressed in milligrams per 100 g of dry weight (mg 100 g^−1^ DW). The chlorophyll content was determined using Equation S4.

### 4.7. Chemical Reagents 

The reagents and solvents used during the investigation were sourced from Sigma Aldrich 99% (St. Louis, MO, USA).

### 4.8. Statistical Analysis

Data were analyzed by two-way ANOVA using InfoStat software (v2020) (Universidad Nacional de Córdoba, Córdoba, Argentina). Tukey’s simultaneous test (*p* ≤ 0.05) was used for means separation.

## 5. Conclusions

The synergistic application of GLU and 6-BAP showed beneficial effects in blueberries, resulting in substantial increases in the photosynthetic pigments, antioxidant defense mechanisms, and number of flower buds, which could result in an increase in yield. The application of both biostimulants could be considered as a promising practice to improve the production, in quantity and quality, of blueberry fruits.

## Figures and Tables

**Table 1:** Effect of the application of the biostimulants glutamate (GLU) and 6-benzylaminopurine (6-BAP) on the number of buds and fruit characteristics of blueberry (*Vaccinium corymbosum* L.) Biloxi.

Treatments		NB	TSS (°Brix)	PDF (mm)	EDF (mm)	FW (g)	TA (% de A. C.)
GLU	0	15.40 ± 0.41 b	13.50 ±0.61b	10.17±0.56 b	14.93 ± 0.87 b	1.69 ± 0.04 b	0.32 ± 0.01 b
	250	16.70 ± 0.43 b	15.17 ±0.47a	10.22 ±0.20 b	14.06 ± 0.56 b	1.73 ± 0.04 b	0.40 ± 0.04 a
	500	18.90 ± 0.54 a	15.50 ±0.37a	11.45 ±0.22 a	16.48 ± 0.61 a	1.95 ± 0.06 a	0.37 ± 0.03 ab
	ANOVA	<0.0001	0.0021	<0.0001	0.0002	<0.0001	0.0366
6-BAP	0	15.46± 0.47 b	14.00±0.77 a	9.43± 0.46 b	12.45 ± 0.46 b	1.64 ± 0.03 b	0.43 ± 0.03 a
	10	17.60 ± 0.63 a	14.92 ±0.45 a	11.32 ±0.25 a	16.62 ± 0.59 a	1.72 ± 0.07 a	0.30 ± 0.02 b
	20	17.90 ± 0.51 a	15.25 ±0.25 a	11.18 ±0.20 a	16.39 ± 0.39 a	1.87 ± 0.04 a	0.36 ± 0.03 ab
	ANOVA	0.0016	0.0748	<0.0001	<0.0001	<0.0001	0.0016
GLU*6-BAP	ANOVA	0.0082	0.0250	0.0002	0.0427	0.0006	0.0007
	CV	7.35	9.01	6.52	8.09	18.21	16.31

Number of buds (NB), total soluble solids (TSS), polar diameter of fruit (PDF), equatorial diameter of fruit (EDF), fruit weight (FW), titratable acidity (TA), variation coefficient (CV). Different letters within columns indicate significant difference (Tukey’s, *p* ≤ 0.05). *n* = 6 ± standard error.

**Table 2:** Effect of the application of the biostimulants glutamate (GLU) and 6-benzylaminopurine (6-BAP) on the content of nonenzymatic antioxidants in blueberry (*Vaccinium corymbosum* L.) fruits.

Treatments		Phenols (mg g^−1^ DW)	Flavonoids (mg g^−1^ DW)	GSH (mmol 100 g^−1^ DW)	Vitamin C (mg 100 g^−1^ FW)	Anthocyanins (mg 100 g^−1^ DW)
GLU	0	40.35 ± 8.13 a	55.38 ± 10.31 b	1.13 ± 0.31 b	12.16 ± 0.17 b	267.15 ± 18.58 b
	250	42.04 ± 6.08 a	58.25 ± 5.07 b	1.22 ± 0.07 ab	12.69 ± 0.18 b	301.68 ± 17.88 a
	500	40.91 ± 5.56 a	64.08 ± 6.39 a	1.31 ± 0.15 a	14.18 ± 0.44 a	311.59 ± 16.67 a
	ANOVA	0.7392	<0.0001	0.0188	<0.0001	0.006
6-BAP	0	42.58 ± 6.93 a	56.63 ± 7.34 b	1.13 ± 0.33 b	12.42 ± 0.27 b	225.22 ± 7.87 c
	10	40.07 ± 6.15 a	56.17 ± 6.69 b	1.22 ± 0.08 ab	13.09 ± 0.32 b	281.08 ± 13.09 b
	20	40.64 ± 6.78 a	64.91 ± 8.02 a	1.30 ± 0.10 a	13.52 ± 0.53 a	374.11 ± 5.97 a
	ANOVA	0.4956	<0.0001	0.0266	0.0039	<0.0001
GLU*6-BAP	ANOVA	0.148	<0.0001	0.009	0.0096	<0.0001
	CV	14.65	7.51	13.87	4.62	12.65

Dry weight (DW), fresh weight (FW), reduced glutathione (GSH), variation coefficient (CV). Different letters within columns indicate significant difference (Tukey’s, *p* ≤ 0.05). *n* = 6 ± standard error.

**Table 3:** Effect of the application of the biostimulants glutamate (GLU) and 6-benzylaminopurine (6-BAP) on nonenzymatic antioxidant content in blueberry (*Vaccinium corymbosum* L.) leaves.

Treatments		Phenols (mg g^−1^ DW)	Flavonoids (mg g^−1^ DW)	GSH (mmol 100 g^−1^ DW)
GLU	0	36.36 ± 6.77 b	38.32 ± 1.67 b	3.19 ± 0.98 b
	250	45.64 ± 4.72 a	41.58 ± 2.79 a	3.72 ± 0.39 a
	500	48.61 ± 2.64 a	40.74 ± 2.88 a	3.17 ± 0.35 b
	ANOVA	<0.0001	0.1639	<0.0001
6-BAP	0	40.9 ± 6.05 b	39.08 ± 2.03 b	3.71 ± 0.31 a
	10	46.52 ± 5.10 a	40.01 ± 3.31 ab	3.04 ± 0.98 c
	20	43.18 ± 9.10 b	41.54 ± 2.54 a	3.33 ± 0.38 b
	ANOVA	0.0008	0.132	<0.0001
GLU*6-BAP	ANOVA	0.0009	<0.0001	<0.0001
	CV	8.48	4.57	6.62

Dry weight (DW), reduced glutathione (GSH), variation coefficient (CV). Different letters within columns indicate significant difference (Tukey’s, *p* ≤ 0.05). *n* = 6 ± standard error.

**Table 4:** Effect of the application of the biostimulants glutamate (GLU) and 6-benzylaminopurine (6-BAP) on photosynthetic pigment content.

Treatments		Chlorophyll *a* (mg 100 g^−1^ FW)	Chlorophyll *b* (mg 100 g^−1^ FW)	Total Chlorophyll (mg 100 g^−1^ FW)
GLU	0	70.99 ± 2.32 c	52.07 ± 3.05 b	123.06 ± 2.15 c
	250	75.29 ± 3.07 b	56.18 ± 5.75 a	131.47 ± 3.11 b
	500	83.95 ± 5,15 a	57.46 ± 2.27 a	141.41 ± 5.99 a
	ANOVA	<0.0001	0.0068	<0.0001
6-BAP	0	75.19 ± 3.95 b	55.92 ± 3.52 a	131.11 ± 2.64 a
	10	77.52 ± 7.16 a	54.46 ± 5.09 a	131.98 ± 3.58 a
	20	77.51 ± 8 a	55.33 ± 6.54 a	132.85 ± 6.16 a
	ANOVA	0.0084	0.6781	0.6814
GLU*6-BAP	ANOVA	<0.0001	<0.0001	<0.0001
	CV	2.89	8.24	4.08

Fresh weight (FW), variation coefficient (CV). Different letters within columns indicate significant difference (Tukey’s, *p* ≤ 0.05). *n* = 6 ± standard error.

**Table 5:** Effect of the application of biostimulants glutamate (GLU) and 6-benzylaminopurine (6-BAP) on enzymatic antioxidant activity in blueberry *(Vaccinium corymbosum* L.) fruits.

Treatments		CAT (U g 100 g^−1^ TP)	PAL (U g 100 g^−1^ TP)	GPX (U g 100 g^−1^ TP)	APX (U g 100 g^−1^ TP)
GLU	0	3.98 ± 1.40 b	0.57 ± 0.10 a	3.98 ± 1.18 b	6.76 ± 1.10 ab
	250	3.34 ± 1.36 b	0.49 ± 0.06 b	3.71 ± 0.86 b	6.92 ± 1.35 a
	500	5.06 ± 0.91 a	0.61 ± 0.16 a	5.1 ± 0.61 a	5.93 ± 0.95 b
	ANOVA	0.0009	0.0025	0.0001	0.0356
6-BAP	0	4.09 ± 1.21 a	0.5 ± 0.07 b	3.76 ± 1.22 b	6.53 ± 1.18 a
	10	4.38 ± 1.55 a	0.55 ± 0.14 ab	4.51 ± 1.14 a	6.47 ± 1.11 a
	20	3.92 ± 1.51 a	0.63 ± 0.11 a	4.52 ± 0.71 a	6.62 ± 1.31 a
	ANOVA	0.5495	0.0012	0.0192	0.9229
GLU*6-BAP	ANOVA	0.0318	0.0272	0.058	0.0725
	CV	27.81	16.04	18.76	17.4

Catalase (CAT), phenylalanine ammonia lyase (PAL), glutathione peroxidase (GPX), ascorbate peroxidase (APX), variation coefficient (CV). Different letters within columns indicate significant difference (Tukey’s, *p* ≤ 0.05). *n* = 6 ± standard error.

**Table 6:** Effect of the application of biostimulants glutamate (GLU) and 6-benzylaminopurine (6-BAP) on the enzymatic activity in blueberry (*Vaccinium corymbosum* L.) leaves.

Treatments		CAT (U g 100 g^−1^ TP)	PAL (U g 100 g^−1^ TP)	GPX (U g 100 g^−1^ TP)	APX (U g 100 g^−1^ TP)
GLU	0	147.03 ± 11.34 b	46.93 ± 6.0 a	460.51 ± 20.86 a	20.33 ± 0.71 b
	250	180.42 ± 6.48 b	51.70 ± 5.74 a	512.97 ± 11.93 a	19.59 ± 0.55 b
	500	247.42 ± 9.09 a	46.93 ± 4.13 a	437.05 ± 5.94 a	27.69 ± 1.46 a
	ANOVA	0.0001	0.2278	0.0729	<0.0001
6-BAP	0	174.77 ± 7.37 b	52.11 ± 5.18 a	492.11 ± 14.52 a	20.03 ± 0.90 b
	10	168.17 ± 15.98 b	50.10 ± 6.68 a	407.29 ± 17.51 b	21.96 ± 1.28 b
	20	231.93 ± 11.87 a	53.14 ± 4.21 a	511.14 ± 18.57 a	25.60 ± 1.49 a
	ANOVA	0.0062	0.8588	0.0071	<0.0001
GLU*6-BAP	ANOVA	0.3328	<0.0001	<0.0001	0.0067
	C.V.	29.26	29.52	19.07	11.83

Catalase (CAT), phenylalanine ammonia lyase (PAL), glutathione peroxidase (GPX), ascorbate peroxidase (APX), variation coefficient (C.V.). Different letters within columns indicate significant difference (Tukey’s, *p* ≤ 0.05). *n* = 6 ± standard error.

**Table 7:** Ion concentration of the nutrient solution used in the different stages of the cultivation of blueberry (*Vaccinium corymbosum* L.) cv. Biloxi.

Phenological Stage	mEq L^−1^								
	CE	pH	NO_3_^−^	NH_4_^+^	H_2_PO_4_^−^	SO_4_^2−^	K^+^	Ca^2+^	Mg^2+^
**Vegetative**	1.1–1.2	5.0–5.5	4	5	1.5	5.5	2.5	2	1.5
**Differentiation Flowering**	0.8–0.9	5.0–5.5	2	2	1.5	5	3.5	2	1.0
**Fruit production**	1.1–1.3	5.0–5.5	3	3	1.5	6	4	2.25	1.25

Electric conductivity (CE), hydrogen potential (pH), nitrate (NO_3_**^−^**), ammonium (NH_4_^+^), phosphoric acid (H_2_PO_4_**^−^**), sulfate (SO_4_^2^**^−^**), potassium (K^+^), calcium (Ca^2+^), magnesium (Mg^2+^).

**Table 8:** Glutamate (GLU) and 6-benzyl amino purine (6-BAP) treatments applied to blueberry (*Vaccinium corymbosum* L.) cv. Biloxi.

Treatment	GLU (mg L^−1^)	6-BAP (mg L^−1^)	Keys
T1 *	0	0	0–0 mg L^−1^
T2	0	10	0–10 mg L^−1^
T3	0	20	0–20 mg L^−1^
T4	250	0	250–0 mg L^−1^
T5	250	10	250–10 mg L^−1^
T6	250	20	250–20 mg L^−1^
T7	500	0	500–0 mg L^−1^
T8	500	10	50–10 mg L^−1^
T9	500	20	500–20 mg L^−1^

(*) Control distilled water.

## Data Availability

Data are available by request to the corresponding author.

## Supplementary Materials

The following supporting information can be downloaded at: https://www.mdpi.com/article/10.3390/plants12122363/s1. **Figure S1.** Calibration curve of total phenols; **Figure S2.** Calibration curve of flavonoids; **Figure S3.** Calibration curve of reduced glutathione; **Figure S4.** Calibration curve of catalase; **Figure S5.** Calibration curve of glutathione peroxidase; **Figure S6.** Calibration curve of phenylalanine ammonium lyase; **Figure S7.** Calibration curve of ascorbate peroxidase; **Equation S1.** Quantification of titratable acidity; **Equation S2.** Quantification of vitamin C; **Equation S3.** Quantification of anthocyanins; **Equation S4.** Determination of photosynthetic pigments.

## References

[1] Loera-Alvarado M. (2017). Aspersión de thidiazuron y ácido giberélico combinado con poda sobre fenología del arándano (*Vaccinium* spp.). Agro Product..

[2] Sarkar D., Kar S.K., Chattopadhyay A., Shikha, Rakshit A., Tripathi V.K., Dubey P.K., Abhilash P.C. (2020). Low Input Sustainable Agriculture: A Viable Climate-Smart Option for Boosting Food Production in a Warming World. Ecol. Indic..

[3] Del Buono D. (2021). Can Biostimulants Be Used to Mitigate the Effect of Anthropogenic Climate Change on Agriculture? It Is Time to Respond. Sci. Total Environ..

[4] Dalal A., Bourstein R., Haish N., Shenhar I., Wallach R., Moshelion M. (2019). Dynamic Physiological Phenotyping of Drought-Stressed Pepper Plants Treated with “Productivity-Enhancing” and “Survivability-Enhancing” Biostimulants. Front. Plant Sci..

[5] Du Jardin P. (2015). Plant Biostimulants: Definition, Concept, Main Categories and Regulation. Sci. Hortic..

[6] European Union (EU) Regulation of the European Parliament and of the Council Laying Down Rules on the Making Available on the Market of EU Fertilizing Products ond Amending Regulations (EC) No. 1069/2009 and (EC) No. 1107/2009 and Repealing Regulation (EC) No. 2003/2003, 2019. https://eur-lex.europa.eu/legal-content/EN/TXT/?uri=OJ:L:2019:170:TOC.

[7] Diario Oficial de la Federación (DOF) NORMA Oficial Mexicana NOM-182-SSA1-2010, Etiquetado de Nutrientes Vegetales. https://www.dof.gob.mx/normasOficiales/4371/salud1a1.htm#:~:text=1.1%20Esta%20norma%20establece%20las,regladores%20de%20crecimiento%20tipo%203.

[8] Panfili I., Bartucca M.L., Marrollo G., Povero G., Del Buono D. (2019). Application of a Plant Biostimulant To Improve Maize (*Zea mays*) Tolerance to Metolachlor. J. Agric. Food Chem..

[9] Albarracín S.L., Baldeón M.E., Sangronis E., Cucufate Petruschina A., Reyes F.G.R. (2016). L-Glutamato: Un aminoácido clave para las funciones sensoriales y metabólicas. Arch. Latinoam. Nutr..

[10] Hassan N.M.K., Marzouk N.M., Fawzy Z.F., Saleh S.A. (2020). Effect of Bio-Stimulants Foliar Applications on Growth, Yield, and Product Quality of Two Cassava Cultivars. Bull. Natl. Res. Cent..

[11] Kong D., Ju C., Parihar A., Kim S., Cho D., Kwak J.M. (2015). Arabidopsis Glutamate Receptor Homolog3.5 Modulates Cytosolic Ca^2+^ Level to Counteract Effect of Abscisic Acid in Seed Germination. Plant Physiol..

[12] Qiu X.-M., Sun Y.-Y., Ye X.-Y., Li Z.-G. (2020). Signaling Role of Glutamate in Plants. Front. Plant Sci..

[13] Mazher A.A.M., Zaghloul S.M., Mahmoud S.A., Siam H.S. (2011). Stimulatory Effect of Kinetin, Ascorbic Acid and Glutamic Acid on Growth and Chemical Constituents of *Codiaeum variegatum* L. Plants. Am.-Eurasian J. Agric. Environ. Sci..

[14] Serna-Rodríguez J.R., Castro-Brindis R., Colinas-León M.T., Sahagún-Castellanos J., Rodríguez-Pérez J.E. (2011). Foliar application of glutamic acid to tomato plants (*Lycopersicon esculentum Mill*.). Rev. Chapingo Ser. Hortic..

[15] Soberanes-Pérez A., Calderón-Zavala G., López-Jiménez A., Alvarado-Raya H.E. (2020). Biorreguladores para la producción de higo bajo condiciones de invernadero. Rev. Fitotec. Mex..

[16] Michard E., Lima P.T., Borges F., Silva A.C., Portes M.T., Carvalho J.E., Gilliham M., Liu L.-H., Obermeyer G., Feijó J.A. (2011). Glutamate Receptor-like Genes Form Ca^2+^ Channels in Pollen Tubes and Are Regulated by Pistil D-Serine. Science.

[17] Wudick M.M., Portes M.T., Michard E., Rosas-Santiago P., Lizzio M.A., Nunes C.O., Campos C., Santa Cruz Damineli D., Carvalho J.C., Lima P.T. (2018). CORNICHON Sorting and Regulation of GLR Channels Underlie Pollen Tube Ca^2+^ Homeostasis. Science.

[18] Yu C., Lv D.G., Qin S.J., Yang L., Ma H.Y., Liu G.C. (2010). Changes in Photosynthesis, Fluorescence and Nitrogen Metabolism of Hawthorn (*Crataegus pinnatifida*) in Response to Exogenous Glutamic Acid. Photosynthetica.

[19] El-Shiekh A.F., Umaharan P. (2014). Effect of Gibberellic Acid, Glutamic Acid and Pollen Grains Extract on Yield, Quality and Marketa-Bility of “khalas” Date Palm Fruits. Acta Hortic..

[20] Kan C.-C., Chung T.-Y., Wu H.-Y., Juo Y.-A., Hsieh M.-H. (2017). Exogenous Glutamate Rapidly Induces the Expression of Genes Involved in Metabolism and Defense Responses in Rice Roots. BMC Genom..

[21] Li Z.-G., Ye X.-Y., Qiu X.-M. (2019). Glutamate Signaling Enhances the Heat Tolerance of Maize Seedlings by Plant Glutamate Receptor-like Channels-Mediated Calcium Signaling. Protoplasma.

[22] Li H., Jiang X., Lv X., Ahammed G.J., Guo Z., Qi Z., Yu J., Zhou Y. (2019). Tomato GLR3.3 and GLR3.5 Mediate Cold Acclimation-Induced Chilling Tolerance by Regulating Apoplastic H_2_O_2_ Production and Redox Homeostasis. Plant Cell Environ..

[23] Yoshida R., Mori I.C., Kamizono N., Shichiri Y., Shimatani T., Miyata F., Honda K., Iwai S. (2016). Glutamate Functions in Stomatal Closure in Arabidopsis and Fava Bean. J. Plant Res..

[24] Cortleven A., Ehret S., Schmülling T., Johansson H. (2019). Ethylene-Independent Promotion of Photomorphogenesis in the Dark by Cytokinin Requires COP1 and the CDD Complex. J. Exp. Bot..

[25] Saini S., Kaur N., Pati P.K. (2021). Phytohormones: Key Players in the Modulation of Heavy Metal Stress Tolerance in Plants. Ecotoxicol. Environ. Saf..

[26] Duarte E. (2022). Regeneración de yemas adventicias en segmentos de hojas y entrenudos de Balfourodendron riedelianum (Engl.) Engl. Colomb. For..

[27] Li Y., Zhang D., Xing L., Zhang S., Zhao C., Han M. (2016). Effect of Exogenous 6-Benzylaminopurine (6-BA) on Branch Type, Floral Induction and Initiation and Related Gene Expression in ‘Fuji’ Apple (*Malus domestica Borkh*). Plant Growth Regul..

[28] Mangena P. (2022). Evolving Role of Synthetic Cytokinin 6-Benzyl Adenine for Drought Stress Tolerance in Soybean (*Glycine max* L.. Merr.). Front. Sustain. Food Syst..

[29] Ramy G.E.-K., Atef M.K.N., Ahmed A.A.E.-S. (2019). The Role of Benzyl Amino Purine and Kinetin in Enhancing the Growth and Flowering of Three Gaillardia Varieties. Alex. J. Agric. Sci..

[30] Burke J.J. (2013). 6-Benzyladenine Enhancements of Cotton Yields. J. Cotton Sci..

[31] Yang D.Q., Luo Y.L., Dong W.H., Yin Y.P., Li Y., Wang Z.L. (2018). Response of Photosystem II Performance and Antioxidant Enzyme Activities in Stay-Green Wheat to Cytokinin. Photosynthetica.

[32] Wang Y., Lu J.W., Ren T., Li P.F., Liu Q.X., Li X.K. (2020). Effects of Exogenous Cytokinin on Photosynthesis, Senescence, and Yield Performance of Inferior Rice Tillers Grown under Different Nitrogen Regimes. Photosynthetica.

[33] Wang J., Wang Y.L., Wang D.Y., Huang J.X., Liu Y.B., Zhu M., Li F.H. (2022). Mitigative Effect of 6-Benzyladenine on Photosynthetic Capacity and Leaf Ultrastructure of Maize Seedlings under Waterlogging Stress. Photosynthetica.

[34] Grimes S.J., Phillips T.D., Hahn V., Capezzone F., Graeff-Hönninger S. (2018). Growth, Yield Performance and Quality Parameters of Three Early Flowering Chia (*Salvia hispanica* L.) Genotypes Cultivated in Southwestern Germany. Agriculture.

[35] Yang H., Tian T., Wu D., Guo D., Lu J. (2019). Prevention and Treatment Effects of Edible Berries for Three Deadly Diseases: Cardiovascular Disease, Cancer and Diabetes. Crit. Rev. Food Sci. Nutr..

[36] Kalt W., Cassidy A., Howard L.R., Krikorian R., Stull A.J., Tremblay F., Zamora-Ros R. (2020). Recent Research on the Health Benefits of Blueberries and Their Anthocyanins. Adv. Nutr..

[37] Wuyang H., Zheng Y., Dajing L., Yanhong M., Jianzhong Z., Zhongquan S. (2018). Antioxidant and Anti-Inflammatory Effects of Blueberry Anthocyanins on High Glucose-Induced Human Retinal Capillary Endothelial Cells. Oxid. Med. Cell. Longev..

[38] Rodríguez-Daza M.-C., Daoust L., Boutkrabt L., Pilon G., Varin T., Dudonné S., Levy É., Marette A., Roy D., Desjardins Y. (2020). Wild Blueberry Proanthocyanidins Shape Distinct Gut Microbiota Profile and Influence Glucose Homeostasis and Intestinal Phenotypes in High-Fat High-Sucrose Fed Mice. Sci. Rep..

[39] Wood E., Hein S., Heiss C., Williams C., Rodriguez-Mateos A. (2019). Blueberries and Cardiovascular Disease Prevention. Food Funct..

[40] Gill K., Kumar P., Negi S., Sharma R., Joshi A.K., Suprun I.I., Al-Nakib E.A. (2023). Physiological Perspective of Plant Growth Regulators in Flowering, Fruit Setting and Ripening Process in Citrus. Sci. Hortic..

[41] Milyaev A., Kofler J., Klaiber I., Czemmel S., Pfannstiel J., Flachowsky H., Stefanelli D., Hanke M.-V., Wünsche J.-N. (2021). Toward Systematic Understanding of Flower Bud Induction in Apple: A Multi-Omics Approach. Front. Plant. Sci..

[42] Agustí M., Reig C., Martínez-Fuentes A., Mesejo C. (2022). Advances in Citrus Flowering: A Review. Front. Plant. Sci..

[43] Ávila J., Salvo S., Muñoz C. (2013). Comparison of Linear Regression Models Considering Heteroscedasticity of Fruits and Flower Buds of Highbush Blueberry Cultivated in Chile. Sci. Hortic..

[44] Kumarihami H.M.P.C., Park H.-G., Kim S.-M., Park J.-I., Lee E.-J., Kim H.L., Kim J.G. (2021). Flower and Leaf Bud Density Manipulation Affects Fruit Set, Leaf-to-Fruit Ratio, and Yield in Southern Highbush ‘Misty’ Blueberry. Sci. Hortic..

[45] El-Metwally I.M., Sadak M.S., Saudy H.S. (2022). Stimulation Effects of Glutamic and 5-Aminolevulinic Acids on Photosynthetic Pigments, Physio-Biochemical Constituents, Antioxidant Activity and Yield of Peanut. Gesunde Pflanz..

[46] Farid M., Farid S., Zubair M., Ghani M.A., Rizwan M., Ishaq H.K., Alkahtani S., Abdel-Daim M.M., Ali S. (2020). Glutamic Acid-Assisted Phytomanagement of Chromium Contaminated Soil by Sunflower (*Helianthus annuus* L.): Morphophysiological and Biochemical Alterations. Front. Plant. Sci..

[47] Zhang Z., Zhang Y., Zhang S., Wang L., Liang X., Wang X., Wu H., Zou H., Zhang C., Wang M. (2022). Foliar Spraying of 6-Benzylaminopurine Promotes Growth and Flavonoid Accumulation in Mulberry (*Morus alba* L.). J. Plant Growth Regul..

[48] Valverde-Miranda D., Díaz-Pérez M., Gómez-Galán M., Callejón-Ferre Á.-J. (2021). Total Soluble Solids and Dry Matter of Cucumber as Indicators of Shelf Life. Postharvest Biol. Technol..

[49] Ariza Flores R., Barrios Ayala A., Herrera García M., Barbosa Moreno F., Michel Aceves A., Otero Sánchez M.A., Alia Tejacal I. (2015). Fitohormonas y bioestimulantes para la floración, producción y calidad de lima mexicana de invierno. Rev. Mex. Cienc. Agrícolas.

[50] Madrid M., Beaudry R. (2020). Small fruits: Raspberries, blackberries, blueberries. Controlled and Modified Atmospheres for Fresh and Fresh-Cut: Produce.

[51] FAO Protocolo de Calidad Para Arándanos Frescos Boletin Oficial No. 31.163. https://www.fao.org/faolex/results/details/en/c/LEX-FAOC071758.

[52] Canli F., Pektas M. (2015). Improving Fruit Size and Quality of Low Yielding and Small Fruited Pear Cultivars with Benzyladenine and Gibberellin Applications. Eur. J. Hortic. Sci..

[53] Milić B., Tarlanović J., Keserović Z., Magazin N., Miodragović M., Popara G. (2018). Bioregulators Can Improve Fruit Size, Yield and Plant Growth of Northern Highbush Blueberry (*Vaccinium corymbosum* L.). Sci. Hortic..

[54] Abdelgadir H.A., Jäger A.K., Johnson S.D., Van Staden J. (2010). Influence of Plant Growth Regulators on Flowering, Fruiting, Seed Oil Content and Oil Quality of *Jatropha Curcas*. S. Afr. J. Bot..

[55] González-Villagra J., Reyes-Díaz M., Alberdi M., Mora M.L., Ulloa-Inostroza E.M., Ribera-Fonseca A.E. (2020). Impact of Cold-Storage and UV-C Irradiation Postharvest Treatments on Quality and Antioxidant Properties of Fruits from Blueberry Cultivars Grown in Southern Chile. J. Soil Sci. Plant Nutr..

[56] Alam M.A., Islam P., Subhan N., Rahman M.M., Khan F., Burrows G.E., Nahar L., Sarker S.D. (2021). Potential Health Benefits of Anthocyanins in Oxidative Stress Related Disorders. Phytochem. Rev..

[57] Ling D.Z., Jian L.L., Hou B.C. (2012). Effects of glutamic acid and TDZ (Thidiazuron) on the fruit coloration and quality of *Litchi chinensis* Sonn. J. Trop. Subtrop. Bot..

[58] Wang L., Wang Z.H., Li Z.Q., Zhu Y.N. (2006). Promotion of L-Glutamic Acid on Anthocyanin Accumulation of Fuji Apples. J. Fruit Sci..

[59] Li B., Zhang X., Duan R., Han C., Yang J., Wang L., Wang S., Su Y., Wang L., Dong Y. (2022). Genomic Analysis of the Glutathione S-Transferase Family in Pear (*Pyrus communis*) and Functional Identification of PcGST57 in Anthocyanin Accumulation. Int. J. Mol. Sci..

[60] Han Jian N.A.U., Shang Gaopan N.A.U., Zhang Binbin J.A.A.S. (2012). Effects of foliar spraying of L-glutamic acid and rhamnose solution on changes of pigment content and physiological properties in leaves of red-leaf peach in summer. J. Nanjing Agric. Univ..

[61] Amin A.A., Gharib F.A.E., El-Awadi M., Rashad E.-S.M. (2011). Physiological Response of Onion Plants to Foliar Application of Putrescine and Glutamine. Sci. Hortic..

[62] Cui Y., De Guo L., Xing Ming H., Wen D. (2015). Changes in Flavonoids Concentration of Hawthorn (*Crataegus pinnatifida*) in Response to Exogenous Amino Acids. J. Hortic. For..

[63] Chen B., Yang H. (2013). 6-Benzylaminopurine alleviates chilling injury of postharvest cucumber fruit through modulating antioxidant system and energy status. J. Sci. Food Agric..

[64] Franzoni G., Cocetta G., Trivellini A., Garabello C., Contartese V., Ferrante A. (2022). Effect of Exogenous Application of Salt Stress and Glutamic Acid on Lettuce (*Lactuca sativa* L.). Sci. Hortic..

[65] Fardus J., Hossain M.S., Fujita M. (2021). Modulation of the Antioxidant Defense System by Exogenous L-Glutamic Acid Application Enhances Salt Tolerance in Lentil (*Lens culinaris Medik*.). Biomolecules.

[66] Qamer Z., Chaudhary M.T., Du X., Hinze L., Azhar M.T. (2021). Review of Oxidative Stress and Antioxidative Defense Mechanisms in *Gossypium hirsutum* L. in Response to Extreme Abiotic Conditions. J. Cotton Res..

[67] Chen J., Wu X., Yao X., Zhu Z., Xu S., Zha D. (2016). Exogenous 6-Benzylaminopurine Confers Tolerance to Low Temperature by Amelioration of Oxidative Damage in Eggplant (*Solanum melongena* L.) Seedlings. Braz. J. Bot..

[68] O’Brien J.A., Daudi A., Butt V.S., Paul Bolwell G. (2012). Reactive Oxygen Species and Their Role in Plant Defence and Cell Wall Metabolism. Planta.

[69] Rajput V.D., Harish, Singh R.K., Verma K.K., Sharma L., Quiroz-Figueroa F.R., Meena M., Gour V.S., Minkina T., Sushkova S. (2021). Recent Developments in Enzymatic Antioxidant Defence Mechanism in Plants with Special Reference to Abiotic Stress. Biology.

[70] Fardus J., Hossain M.S., Fujita M. (2021). Potential Role of L-Glutamic Acid in Mitigating Cadmium Toxicity in Lentil (*Lens culinaris Medik*.) through Modulating the Antioxidant Defence System and Nutrient Homeostasis. Not. Bot. Horti Agrobot. Cluj Napoca.

[71] Teixeira W.F., Fagan E.B., Soares L.H., Umburanas R.C., Reichardt K., Neto D.D. (2017). Foliar and Seed Application of Amino Acids Affects the Antioxidant Metabolism of the Soybean Crop. Front. Plant. Sci..

[72] Astaneh R.K., Bolandnazar S., Nahandi F.Z., Oustan S. (2018). Effect of Selenium Application on Phenylalanine Ammonia-Lyase (PAL) Activity, Phenol Leakage and Total Phenolic Content in Garlic (*Allium sativum* L.) under NaCl Stress. Inf. Process. Agric..

[73] QiaoZhen L., Ben X., YanLi S., WeiRong X., HongJun D. (2019). Effects of exogenous 6-BA on anthocyanin content and expression of related genes in grape berry. Nat. Sci. Ed..

[74] Chen J.-Y., Wen P.-F., Kong W.-F., Pan Q.-H., Zhan J.-C., Li J.-M., Wan S.-B., Huang W.-D. (2006). Effect of Salicylic Acid on Phenylpropanoids and Phenylalanine Ammonia-Lyase in Harvested Grape Berries. Postharvest Biol. Technol..

[75] Capocasa F., Scalzo J., Mezzetti B., Battino M. (2008). Combining Quality and Antioxidant Attributes in the Strawberry: The Role of Genotype. Food Chem..

[76] Padayatty S.J., Daruwala R., Wang Y., Eck P.K., Song J., Koh W.S., Levine M. (2001). Vitamina C: De las acciones moleculares a la ingesta óptima. Manual de Antioxidantes.

[77] Yu Z., Dahlgren R.A. (2000). Evaluation of Methods for Measuring Polyphenols in Conifer Foliage. J. Chem. Ecol..

[78] Arvouet-Grand A., Vennat B., Pourrat A., Legret P. (1994). Standardization of propolis extract and identification of principal constituents. J. Pharm. Belg..

[79] Xue T., Hartikainen H., Piironen V. (2001). Antioxidative and Growth-Promoting Effect of Selenium on Senescing Lettuce. Plant Soil.

[80] Giusti M.M., Wrolstad R.E. (2001). Characterization and Measurement of Anthocyanins by UV-Visible Spectroscopy. Curr. Protoc. Food Anal. Chem..

[81] Dhindsa R.S., Plumb-Dhindsa P.L., Reid D.M. (1982). Leaf Senescence and Lipid Peroxidation: Effects of Some Phytohormones and Scavengers of Free Radicals and Singlet Oxygen. Physiol. Plant..

[82] Flohé L., Günzler W.A. (1984). Assays of Glutathione Peroxidase. Methods Enzym..

[83] Sykłowska-Baranek K., Pietrosiuk A., Naliwajski M.R., Kawiak A., Jeziorek M., Wyderska S., Lojkowska E., Chinou I. (2012). Effect of L-Phenylalanine on PAL Activity and Production of Naphthoquinone Pigments in Suspension Cultures of Arnebia Euchroma (Royle) Johnst. Vitr. Cell. Dev. Biol. Plant.

[84] Elavarthi S., Martin B., Sunkar R. (2010). Spectrophotometric assays for antioxidant enzymes in plants. Plant Stress Tolerance: Methods and Protocols.

[85] Arnon D.I. (1949). Copper Enzymes in Isolated Chloroplasts: Polyphenoloxidase in *Beta vulgaris*. Plant Physiol..

[86] Munira S., Hossain M., Zakaria M., Ahmed J., Islam M. (2015). Evaluation of Potato Varieties against Salinity Stress in Bangladesh. Int. J. Plant Soil Sci..

