# Supplementary material for: Effect of Glutamic Acid and 6-benzylaminopurine on Flower Bud Biostimulation, Fruit Quality and Antioxidant Activity in Blueberry
Source: Plants (Basel). 2023 Jun 18;12(12):2363. doi: 10.3390/plants12122363 (PMC10301510; doi:10.3390/plants12122363)
Supplement: Supplementary file 1 [file plants-12-02363-s001.zip › plants-2402089-supplementary.pdf]

Curves of calibration

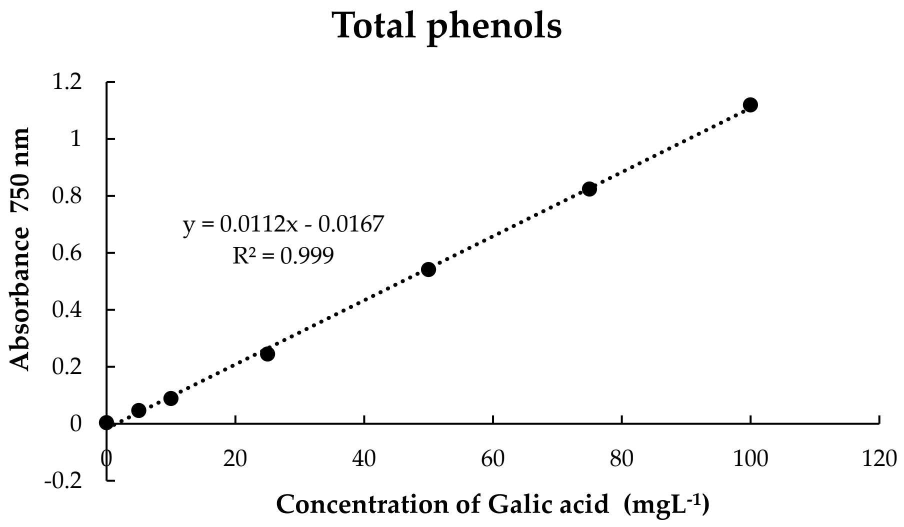

Figure S1. Calibration curve of total phenols. The exponential equation was obtained by modeling the absorbance values at 750 nm versus the concentration of gallic acid (mg L^{-1}).

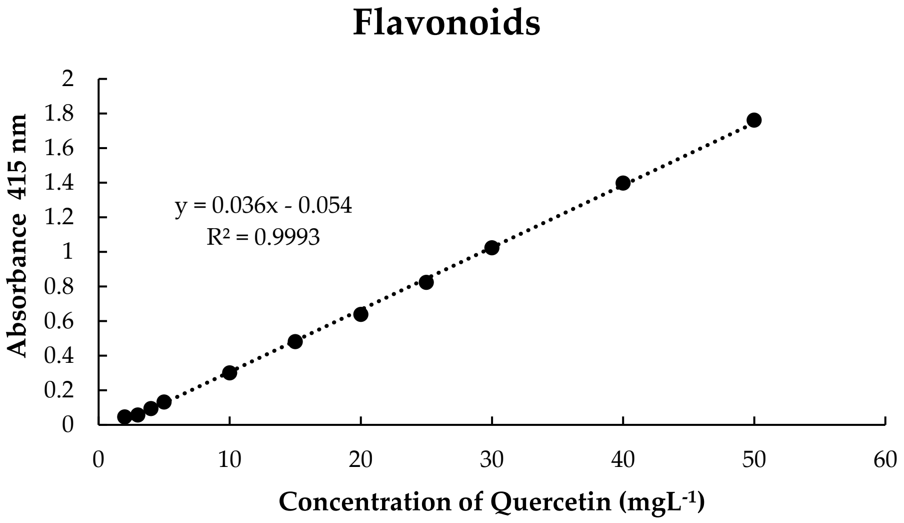

Figure S2. Calibration curve of flavonoids. The exponential equation was obtained by modeling the absorbance values at 415 nm versus the concentration of quercetin (mg L^{-1}).

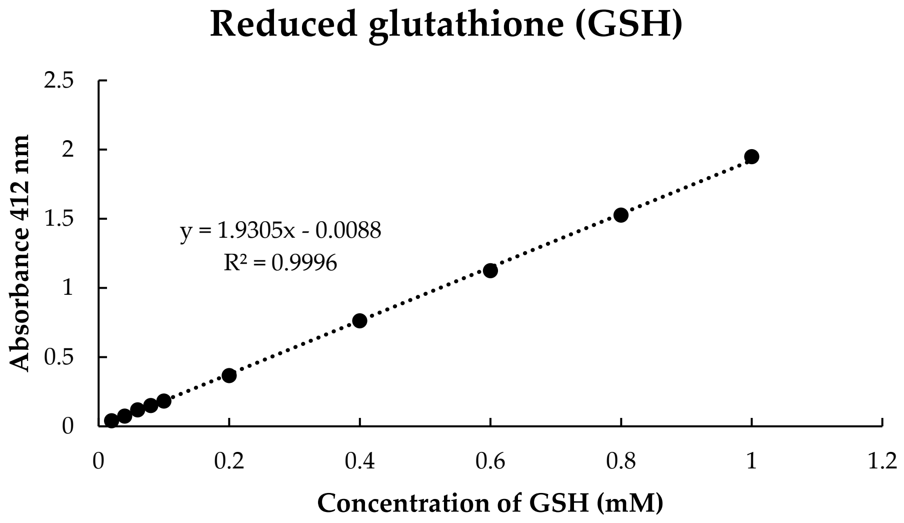

Figure S3. Calibration curve of reduced glutathione. The exponential equation was obtained by modeling the absorbance values at 412 nm versus the concentration of reduced glutathione (mM).

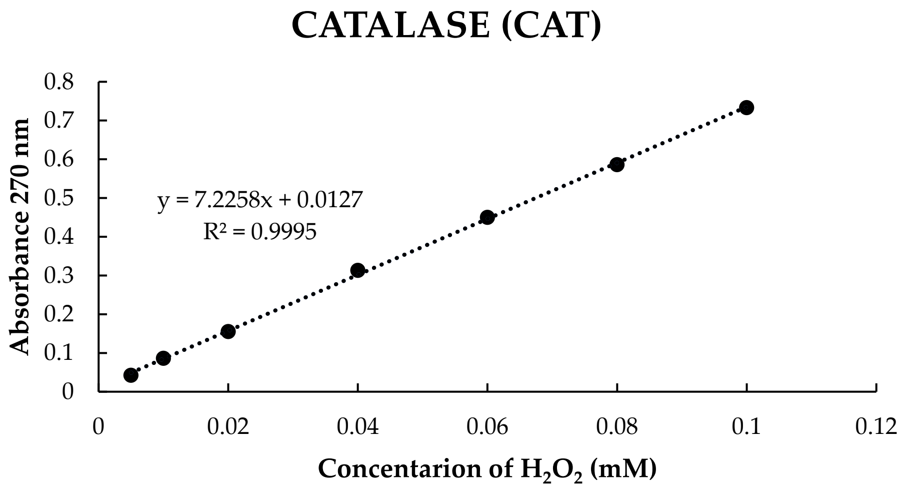

Figure S4. Calibration curve of catalase. The exponential equation was obtained by modeling the absorbance values at 270 nm versus the concentration of hydrogen peroxide (mM).

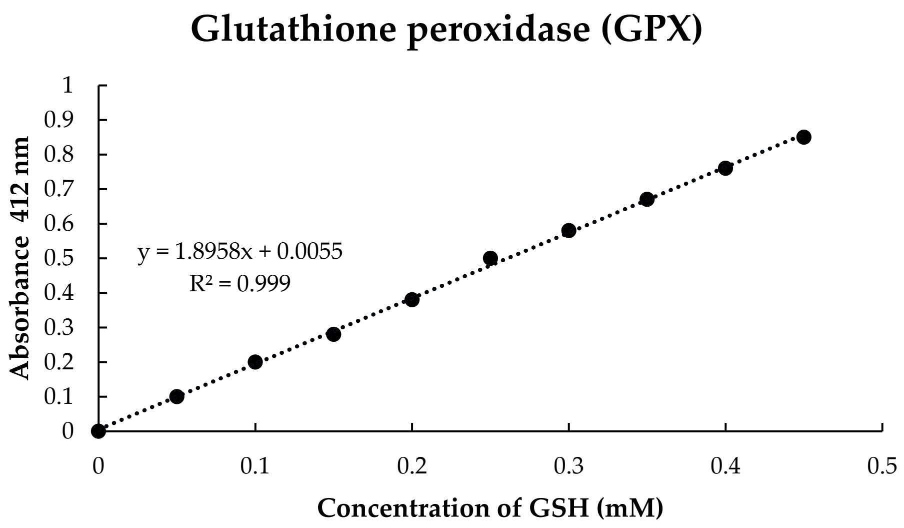

Figure S5. Calibration curve of glutathione peroxidase. The exponential equation was obtained by modeling the absorbance values at 412 nm versus the concentration of reduced glutathione (mM).

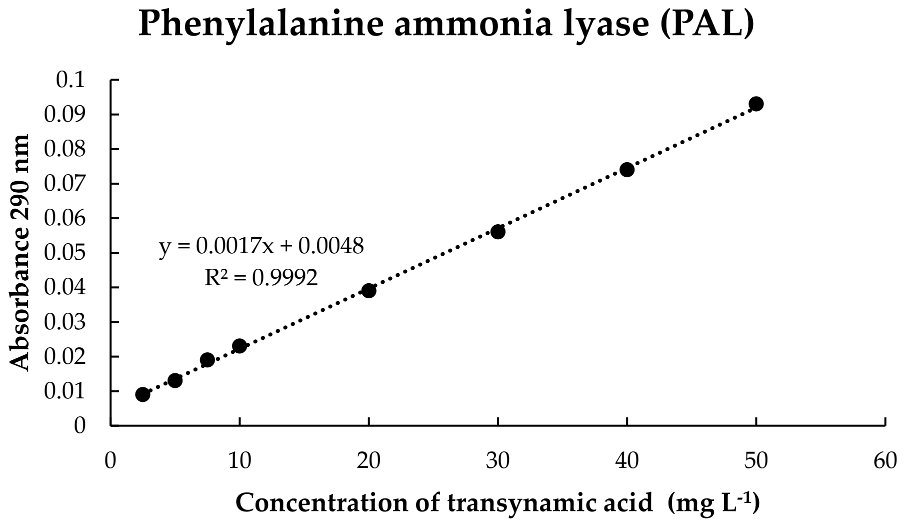

Figure S6. Calibration curve of phenylalanine ammonium lyase. The exponential equation was obtained by modeling the absorbance values at 290 nm versus the concentration of transynamic acid (mg L^{-1}).

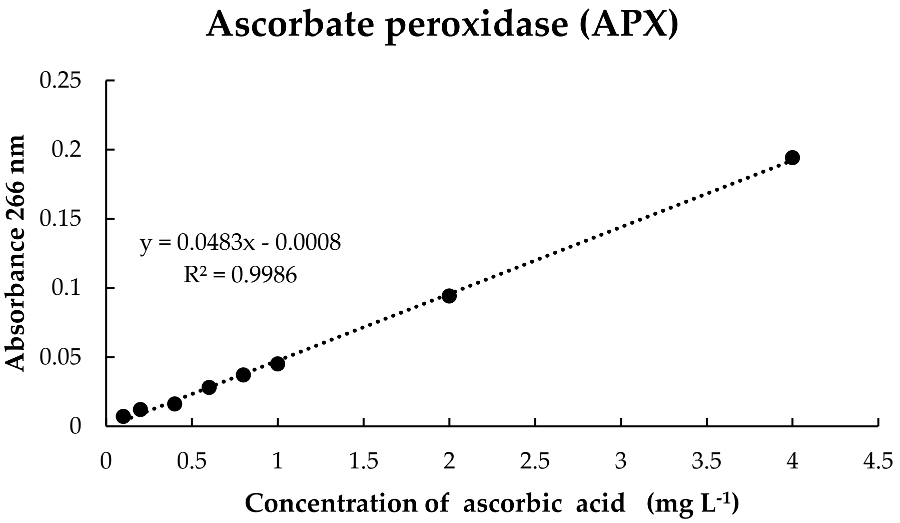

Figure S7. Calibration curve of ascorbate peroxidase. The exponential equation was obtained by modeling the absorbance values at 266 nm versus the concentration of ascorbic acid (mg L^{-1})

$$\% \, citric \, acid = \frac{(VNaOH * meq \, citric \, acid * 100)}{sample \, volume}$$

Equation S1. Quantification of titratable acidity. Where Na OH = sodium hydroxide, VNaOH=volume spent of sodium hydroxide, NNaOH=normality of sodium hydroxide, meq citric acid= 0.064

$$Vitamin \, C = \frac{(mL \, spent \, of \, 2{,}6 \, dichlorophenolindophenol * 0.088 * total \, volume * 100)}{aliquot \, volume * sample \, weight}$$

Equation S2. Quantification of vitamin C, by the titration method with 2,6 dichlorophenolindophenol.

$$\frac{A * MW * DF * 10^3}{\varepsilon * 1}$$

Equation S3. Quantification of anthocyanins. where A= ($A_{520nm \, pH1}$–$A_{700nm \, pH1}$) –($A_{520nm \, pH4.5}$–$A_{700nm \, pH4.5}$); MW (molecular weight) = 449.2 g mol^{-1} for cyanidin-3-glucoside; DF = dilution factor established in D; 1 = path length in cm; ε = 26,900 molar extinction coefficient, in $L*mol^{-1}*cm^{-1}$, for cyanidin-3-glucoside; and 10^3 = factor for conversion from g to mg.

$$\text{Chlorophyll a} = 25.38 * A_{663} + 3.64 * A_{645}$$
$$\text{Chlorophyll b} = 30.38 * A_{645} - 6.58 * A_{663}$$
$$Total \, chlorophyll = 18.8 * A_{663} + 34.02 * A_{645}$$

Equation S4. Determination of photosynthetic pigments.
